# Association of early postoperative serum magnesium with acute kidney injury after cardiac surgery

**DOI:** 10.1080/0886022X.2023.2170244

**Published:** 2023-02-02

**Authors:** Chao Xiong, Sheng Shi, Liang Cao, Hongbai Wang, Lijuan Tian, Yuan Jia, Min Zeng, Jianhui Wang

**Affiliations:** a Department of Anesthesiology, National Center of Cardiovascular Diseases, Fuwai Hospital, Chinese Academy of Medical Sciences and Peking Union Medical College, Beijing, China; b Center for Pediatric Cardiac Surgery, National Center of Cardiovascular Diseases, Fuwai Hospital, Chinese Academy of Medical Sciences and Peking Union Medical College, Beijing, China

**Keywords:** Acute kidney injury, cardiac surgery, magnesium, eICU

## Abstract

**Introduction:**

Dysmagnesemia has been demonstrated to be involved in the pathophysiology of kidney diseases and is common in cardiac surgical patients. It remains unknown whether changes of serum magnesium after cardiac surgery affect AKI. We aimed to investigate the association of early postoperative magnesium with cardiac surgery-associated AKI in adults.

**Methods:**

We conducted a multicenter retrospective cohort study involving patients who underwent cardiac surgery in the eICU Collaborative Research Database between 2014 and 2015. AKI within 7 days after surgery was defined using both serum creatinine and urine output criteria of Kidney Disease Improving Global Outcomes definition. Postoperative AKI was analyzed using multivariable logistic regression with early postoperative serum magnesium measured within the first 24 h after surgery as a continuous variable and categorically by quartiles.

**Results:**

Postoperative AKI was identified in 3498 of 6124 (57.1%) patients receiving cardiac surgery. The median (25th–75th percentiles) early postoperative serum magnesium level of the study population was 2.3 (2.0–2.7) mg/dL. Higher serum magnesium level was associated with a higher risk of developing postoperative AKI (adjusted odds ratio (OR), 1.46 per 1 mg/dL increase; 95% confidence interval (CI), 1.31–1.62; *p*<.001). The multivariable-adjusted ORs (95% CIs) of postoperative AKI across increasing quartiles of serum magnesium were 1.00 (referent), 1.11 (0.95–1.29), 1.30 (1.12–1.52), and 1.72 (1.47–2.02) (*p* for trend <.001).

**Conclusions:**

These data demonstrate a significantly higher incidence of AKI in patients with higher early postoperative serum magnesium who underwent cardiac surgery.

## Introduction

More than two million cardiac surgeries are performed globally each year [1]. AKI is a common complication in the postoperative period and its incidence can be as high as 81.2% defined by both serum creatinine and urine output criteria in cardiac surgical population [2]. Approximately, 1–5% of patients undergoing cardiac surgery require renal replacement therapy (RRT) after developing AKI, leading to three- to eight-fold higher risk of death [3]. The underlying causes of cardiac surgery-associated AKI are multifactorial and incompletely understood. Pathological processes including oxidative stress, inflammation, kidney ischemia, hypovolemia, vasodilatation, and decreased cardiac output are all involved in the development of postoperative AKI [3,4]. However, there are only supportive care but no specific treatments for AKI after its development. Therefore, identification of patients at risk of having AKI is important for targeting modifiable perioperative factors to reduce AKI and improving outcomes.

Magnesium is important for maintaining normal functions of the heart, brain, and skeletal muscles [5]. Alterations in serum magnesium level are common among critically ill patients and associated with the development of several different diseases [5–10]. In cardiac surgery patients, low serum magnesium in the perioperative period is associated with higher incidence of postoperative atrial fibrillation (POAF) and magnesium supplementation is widely used for prevention and treatment of hypomagnesemia and POAF [11,12].

Recently, magnesium has been found to play an important role in the pathophysiology of kidney diseases. Lower total serum magnesium level has been associated with higher risk of incident CKD in the general population and higher risk of CKD progression in those with pre-existing CKD [13,14]. In patients undergoing cardiac surgery, two retrospective cohort studies showed that lower preoperative serum magnesium level was associated with higher risk of AKI [15,16]. In addition, magnesium replacement therapy for patients with postoperative hypomagnesemia decreased the incidence of cardiac surgery-associated AKI in a small randomized controlled trial [17]. However, the association of early postoperative serum magnesium level with AKI in patients having cardiac surgery has not been investigated yet.

We hypothesized that dysmagnesemia after cardiac surgery contributes to the development of AKI. Therefore, we conducted a retrospective cohort study to determine the association between early postoperative total serum magnesium level and AKI in cardiac surgery patients.

## Methods and materials

### Study design

This was a retrospective study of patients admitted into cardiac surgical intensive care units (ICUs) in the eICU Collaborative Research Database (eICU) database, using deidentified and publicly available data. The eICU (version 2.0) is a large multicenter database of a random sample of patients admitted to 291 ICUs in 208 hospitals in the United States from 2014 to 2015. The eICU database was released under the Health Insurance Portability and Accountability Act (HIPAA) safe harbor provision (HIPAA certification no. 1031219-2), and the ethics approval of eICU was not applicable. This study is reported in accordance with the STrengthening the Reporting of Observational studies in Epidemiology (STROBE) statement. Our plan for statistical analysis was made before accessing the data in the eICU database.

### Study cohort

Patients were included if they were age 18 years or older, underwent coronary artery bypass graft (CABG) surgery, valve replacement or repair, or combined CABG and valvular procedures. If patients had multiple cardiac surgeries, only the first index surgical admission was included. Patients were excluded for the following reasons: (1) unclear AKI status during the first 7 days after surgery, (2) received hemodialysis or peritoneal dialysis before surgery, had end-stage renal disease, or baseline serum creatinine level greater than 4 mg/dL, (3) no serum magnesium measurements during the first 24 h after surgery, and (4) incomplete datasets.

### Data extraction

Data were extracted from the eICU database using structured query language. The following demographic and clinical data were collected: age, sex, ethnicity, body mass index, admission type, surgery type, and disease severity scores (Sequential Organ Failure Assessment (SOFA) and the Acute Physiology and Chronic Health Evaluation (APACHE) IV). Diagnosed via the International Classification of Diseases codes, the following 7 comorbidities were considered: congestive heart failure (CHF), peripheral vascular disease, hypertension, chronic pulmonary disease, diabetes, stroke, and CKD. Laboratory measurements of baseline hemoglobin and creatinine were captured as the most recent values within 7 days before surgery. If preoperative baseline creatinine was unavailable, the first serum creatinine values after surgery were considered as baseline. Baseline eGFR was calculated by CKD Epidemiology Collaboration eGFR equation. In addition, medication information of magnesium supplementation that started at postoperative day 1 was extracted.

### Exposure

Early postoperative total serum magnesium concentration was the primary exposure of the present analysis and analyzed as a continuous variable and categorically by quartiles. Early postoperative serum magnesium was defined as the first measured value within 24 h after cardiac surgery.

### Outcomes

The primary outcome was AKI within 7 days after cardiac surgery. The diagnosis of AKI was based on the Kidney Disease Improving Global Outcomes definition using both serum creatinine criteria and urine output [18]. The secondary outcomes included new-onset postoperative RRT within 30 days after surgery and in-hospital mortality.

### Statistical analyses

The data analysis plan was reviewed by all authors in April 2022 before data extraction. Continuous variables were described as medians (25th–75th percentiles) and categorical variables as total numbers and percentages. Baseline and postoperative clinical characteristics were compared using Cochran–Armitage test, Cuzick’s test, and post-estimation trend test based on logistic regression to test the trend across quartiles of early postoperative serum levels of magnesium.

The association of early postoperative serum levels of magnesium as a continuous variable and a four-level categorical variable using quartiles with both primary and secondary outcomes were assessed by unadjusted and adjusted logistic regression. Potential confounding variables adjusted in the logistic regression model included age, sex, ethnicity (white, African American, unknown, or other), body mass index, admission type (non-elective or elective), surgery type (CABG only, valve only, or combined), CHF, peripheral vascular disease, hypertension, chronic pulmonary disease, diabetes, stroke, CKD, baseline hemoglobin, baseline serum creatinine, baseline eGFR, SOFA score, APACHE IV score, and magnesium supplementation. In addition, splines that adjusted by the same covariates in the logistic regression model were used to visualize the association between serum magnesium as a continuous variable and the outcomes.

Several sensitivity analyses were undertaken to test the robustness of our findings. First, we assessed the association between serum magnesium and postoperative AKI using Cox proportional hazard regression models. Second, we performed additional analysis only using the serum creatinine criteria to categorize AKI given that change in urine output is less frequently used for the diagnosis of AKI [19]. Third, patients with incomplete datasets were also included in the sensitivity analysis and missing values were imputed using multiple imputation. The AKI analysis was replicated after multiple imputation to mitigate the bias introduced by missing data. Fourth, we assessed the association between the change of serum magnesium during the perioperative period (ΔMg) and AKI. ΔMg was defined as the difference between the first serum magnesium measurements within 24 h after surgery and preoperative serum magnesium values. Last, the association between preoperative serum magnesium and AKI was evaluated to determine the relationship between preexisting dysmagnesemia before surgical intervention and new-onset postoperative AKI. Preoperative serum magnesium was defined as the most recent value measured within 7 days before surgery. Patients were excluded from the last two sensitivity analyses if preoperative serum magnesium values were lacking.

Three *post hoc* sensitivity analyses were conducted during the peer review process. First, preoperative and postoperative use of loop diuretics was added to the multivariable logistic model two extra covariates to evaluate whether the association between serum magnesium and postoperative AKI was affected by diuretic use. Preoperative use of loop diuretics was defined as receiving any loop diuretic within 7 days before surgery while postoperative exposure was defined as administration of any loop diuretic after surgery but before the first postoperative measurement of serum magnesium. Second, we changed the definition of baseline serum creatinine as only preoperative serum creatinine values were used to define baseline and patients without available preoperative serum creatinine measurement were excluded from the analysis. Third, early postoperative serum potassium was considered as an additional confounding variable and added to the multivariable logistic regression model as a continuous variable. Early postoperative serum potassium was defined as the first serum potassium level measured within 24 h after surgery.

The potential effect modifications of the association of serum magnesium and AKI by age, sex, ethnicity, admission type, surgery type, preexisting CHF, hypertension, diabetes, CKD, baseline eGFR, and magnesium supplementation were assessed by subgroup analyses and interaction tests. Logistic regression models were fit for subgroup analyses and *p* values less than .10 for interaction were considered significant. As an exploratory analysis, we also evaluated the association between magnesium supplementation and AKI development according to early postoperative serum magnesium quartile by multivariable logistic regression.

The outcomes in this study were all dichotomous, and the associations of serum magnesium with the outcomes were described as odds ratios (ORs) or hazard ratios (HRs) and their 95% confidence intervals (CIs). All tests were two-sided, and a *p* value <.05 was considered to indicate statistical significance. All statistical analyses were performed using R software version 4.1.2 (R Foundation for Statistical Computing, Vienna, Austria).

## Results

### Study cohort description

**Figure 1.**
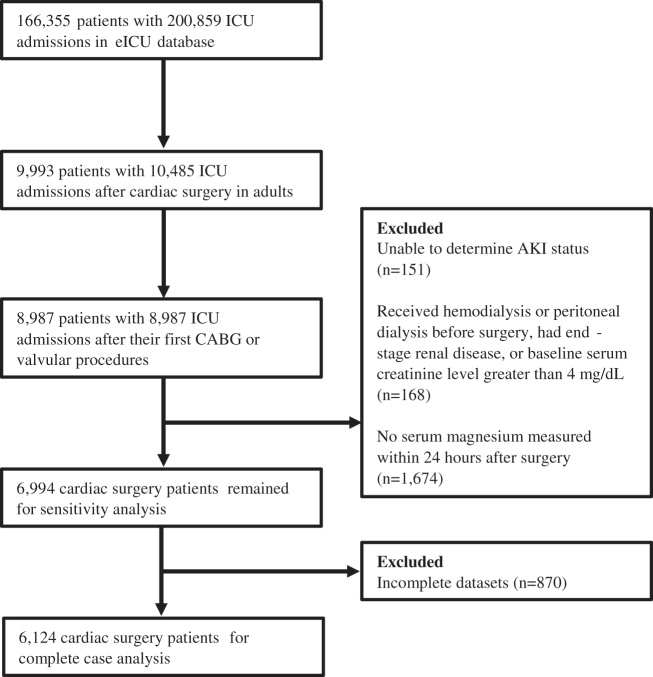
Study cohort flowchart.

A total of 9993 adult patients with 10,485 cardiac surgeries from the eICU database were considered for this study. Among 9993 cardiac surgery patients, we identified 6124 patients from 71 hospitals according to the inclusion and exclusion criteria and only their first index cardiac surgeries with complete datasets were considered in the complete case analysis (Figure 1). The median [25th–75th percentiles] serum magnesium level of the study population was 2.3 [2.0–2.7] mg/dL. Serum magnesium in the early postoperative period was not normally distributed in the study cohort and its distribution is presented in Supplemental Figure 1. Baseline clinical characteristics of the four patient groups, stratified by quartiles of early postoperative serum magnesium level, are shown in Table 1. Individuals with higher serum magnesium tended to be younger, less likely to be men, and more likely to have combined surgery. They also tended to have lower prevalence of comorbidities including CHF, hypertension, and diabetes. In addition, there was a clear upward trend from the lowest to the highest serum magnesium quartile regarding baseline serum creatinine, SOFA score, and APACHE IV score. Lastly, subjects with higher serum magnesium tended to have lower baseline hemoglobin and lower prevalence of magnesium supplementation.

**Table 1.** Baseline and clinical characteristics according to early postoperative serum magnesium quartile.

Characteristic	Early postoperative serum magnesium quartiles, mg/dL				*p* Trend
	Q1 (<2.0 mg/dL) *N* = 1386	Q2 (2.0 to <2.3 mg/dL) *N* = 1522	Q3 (2.3 to <2.7 mg/dL) *N* = 1675	Q4 (≥2.7 mg/dL) *N* = 1541	
Age	69 (60–78)	68 (59–76)	67 (60–75)	68 (60–75)	<.001
Male sex	927 (66.9)	1082 (71.1)	1188 (70.9)	964 (62.6)	.011
Body mass index (kg/m^2^)	28.1 (24.9–32.5)	29.0 (25.5–33.5)	29.2 (25.6–33.5)	28.6 (24.9–33.3)	.125
Ethnicity, *n* (%)					.313
White	1158 (83.6)	1263 (83.0)	1430 (85.4)	1277 (82.9)	
African American	102 (7.4)	96 (6.3)	106 (6.3)	94 (5.7)	
Unknown/others	126 (9.1)	163 (10.7)	139 (8.3)	170 (11.4)	
Admission type					.603
Non-elective	1299 (93.7)	1420 (93.3)	1574 (94.0)	1448 (94.0)	
Elective	87 (6.3)	102 (6.7)	101 (6.0)	93 (6.0)	
Surgery type					<.001
CABG only	765 (55.2)	832 (54.7)	974 (58.2)	785 (50.9)	
Valve only	527 (38.0)	516 (33.9)	515 (30.8)	537 (34.9)	
Combined (CABG + valve)	94 (6.8)	174 (11.4)	186 (11.1)	219 (14.2)	
Comorbidities					
Congestive heart failure	285 (20.6)	257 (16.9)	227 (13.6)	259 (16.8)	.001
Peripheral vascular disease	95 (6.9)	87 (5.7)	101 (6.0)	78 (5.1)	.069
Hypertension	921 (66.5)	1003 (65.9)	1086 (64.8)	966 (62.7)	.025
Chronic pulmonary disease	207 (14.9)	186 (12.2)	214 (12.8)	205 (13.3)	.303
Diabetes	505 (36.4)	499 (32.8)	536 (32.0)	461 (29.9)	<.001
Stroke	88 (6.4)	92 (6.0)	94 (5.6)	105 (6.8)	.721
Chronic kidney disease	175 (12.6)	175 (11.5)	154 (9.2)	191 (12.4)	.428
Laboratory data					
Baseline serum creatinine (mg/dL)	0.8 (0.7–1.1)	0.9 (0.7–1.1)	0.9 (0.7–1.1)	0.9 (0.7–1.2)	<.001
Baseline eGFR (mL/min/1.73 m^2^)	90 (70–112)	89 (69–107)	88 (69–106)	82 (62–103)	<.001
Baseline hemoglobin (g/dL)	9.4 (8.1–10.8)	9.3 (8.1–10.6)	9.5 (8.2–10.7)	9.1 (8.0–10.4)	.002
SOFA score	5 (3–7)	6 (4–8)	6 (4–8)	6 (5–8)	<.001
APACHE IV score	55 (43–69)	54 (43–69)	56 (45–71)	55 (45–69)	.024
Magnesium supplementation	370 (26.7)	219 (14.4)	131 (7.8)	73 (4.7)	<.001

APACHE: acute physiology and chronic health evaluation; CABG: coronary artery bypass grafting; IQR: interquartile range; SOFA: sequential organ failure assessment.

Data are *n* (percentage) or median (25th–75th percentiles).

### Postoperative AKI

**Figure 2.**
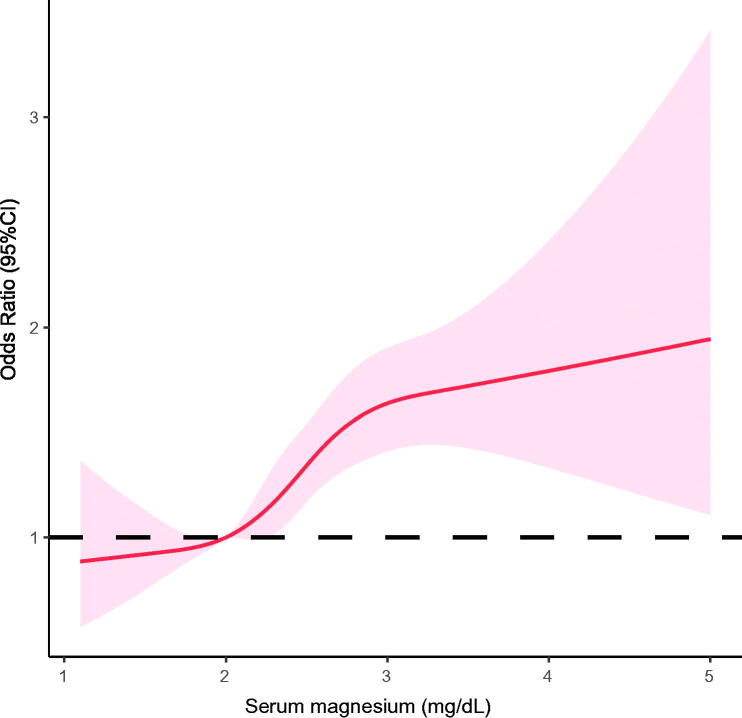
Adjusted splines for the association of early postoperative serum magnesium with acute kidney injury after cardiac surgery. Splines adjusted for age, sex, ethnicity, body mass index, admission type, surgery type, comorbidities, baseline hemoglobin, baseline serum creatinine, baseline eGFR, SOFA score, APACHE IV score, and magnesium supplementation.

The criteria for any-stage postoperative AKI were met by 3498 of 6124 patients (57.1%) in the study population. The incidences of AKI in subjects in the lowest (Q1) to the highest serum magnesium category (Q4) were in 50.6%, 53.4%, 58.4%, and 65.3% (*p* for trend <.001). As a continuous variable, higher serum magnesium was associated with higher risk of developing postoperative AKI (unadjusted OR, 1.52; 95% CI, 1.38–1.68; *p*< .001; Table 2). In a multivariable logistic regression model, this relationship remained significant as per 1 mg/dL increase in serum magnesium level was associated with a 46% higher risk of AKI (adjusted OR, 1.46; 95% CI, 1.31–1.62; *p*< .001; Table 2). When considered as a categorical variable, there was a clear upward trend toward higher incidence of AKI for increasing serum magnesium quartiles (*p* for trend <.001). Adjusted for covariates, subjects in the top serum magnesium quartile (Q4) had a 72% higher risk of AKI development compared with those in the lowest quartile (Q1) (adjusted OR, 1.72; 95% CI, 1.47–2.02; *p* for trend <.001; Table 2). Using restricted cubic spline, we showed that there was a monotonic increase in the risk of having any-stage AKI with increasing serum magnesium level (Figure 2).

**Table 2.** Association of early postoperative serum magnesium with acute kidney injury after cardiac surgery.

Model	Odds ratio (95% confidence interval) per 1 mg/dL increase in serum magnesium	*p* Value	Odds ratio (95% confidence interval)				*p* Trend
			Q1 (<2.0 mg/dL)	Q2 (2.0 to <2.3 mg/dL)	Q3 (2.3 to <2.7 mg/dL)	Q4 (≥2.7 mg/dL)	
*N* events/*N* participants	3498/6124	N/A	701/1386	812/1522	978/1675	1007/1541	N/A
Univariable	1.52 (1.38–1.68)	<.001	Referent	1.12 (0.97–1.29)	1.37 (1.19–1.58)	1.84 (1.59–2.14)	<.001
Multivariable	1.46 (1.32–1.62)	<.001	Referent	1.11 (0.95–1.29)	1.30 (1.12–1.52)	1.72 (1.47–2.02)	<.001

Odds ratios (95% confidence intervals) by multivariable logistic regression models were adjusted for age, sex, ethnicity, body mass index, admission type, surgery type, comorbidities, baseline hemoglobin, baseline serum creatinine, baseline eGFR, SOFA score, APACHE IV score, and magnesium supplementation.

### Associations between serum magnesium and secondary outcomes

Of 6124 cardiac surgery patients, the overall incidence of new-onset postoperative RRT was 2.3% (141/6124). Each 1 mg/dL increase of early postoperative serum magnesium level was associated with an adjusted OR of 1.60 (95% CI, 1.17–2.19; *p* = .003; Table 3) for *de novo* postoperative RRT. In categorical analyses, the incidence of new-onset postoperative RRT was significantly higher in patients in the highest quartile (Q4) of serum magnesium than in those in the lowest (Q1) (Q4 vs. Q1, adjusted OR, 2.15; 95% CI, 1.20–3.86; *p* for trend=.013; Table 3).

**Table 3.** Association of early postoperative serum magnesium with secondary outcomes.

Outcome	Model	Odds ratio (95% confidence interval) per 1 mg/dL increase in serum magnesium	*p* Value	Odds ratio (95% confidence interval)				*p* Trend
				Q1 (<2.0 mg/dL)	Q2 (2.0 to <2.3 mg/dL)	Q3 (2.3 to <2.7 mg/dL)	Q4 (≥2.7 mg/dL)	
New-onset postoperative renal replacement therapy	*N* events/*N* participants	141/6124	N/A	21/1386	29/1522	29/1675	62/1541	N/A
	Univariable	1.77 (1.37–2.29)	<.001	Referent	1.26 (0.72–2.22)	1.15 (0.65–2.01)	2.72 (1.65–4.49)	<.001
	Multivariable	1.60 (1.17–2.19)	.003	Referent	1.24 (0.66–2.33)	1.11 (0.59–2.10)	2.15 (1.20–3.86)	.013
In-hospital mortality	*N* events/*N* participants	129/6124	N/A	20/1386	34/1522	34/1675	41/1541	N/A
	Univariable	1.53 (1.15–2.03)	.003	Referent	1.56 (0.89–2.72)	1.42 (0.81–2.47)	1.87 (1.09–3.20)	.043
	Multivariable	1.50 (1.08–2.07)	.014	Referent	1.43 (0.79–2.58)	1.27 (0.70–2.31)	1.61 (0.90–2.90)	.425

Odds ratios (95% confidence intervals) by multivariable logistic regression models were adjusted for age, sex, ethnicity, body mass index, admission type, surgery type, comorbidities, baseline hemoglobin, baseline serum creatinine, baseline eGFR, SOFA score, APACHE IV score, and magnesium supplementation.

One hundred and twenty-nine of 6124 patients (2.1%) died during their hospital stay. In a multivariable logistic regression model, each 1 mg/dL increase of serum magnesium level was associated with a 50% higher risk of in-hospital mortality (adjusted OR, 1.50; 95% CI, 1.08–2.07; *p* = .014; Table 3). However, we observed no clear upward trend from the lowest to the highest quartile of serum magnesium level regarding in-hospital mortality (*p* for trend=.425; Table 3).

### Sensitivity analyses

Several sensitivity analyses were conducted to test the robustness of our main findings regarding the association between serum magnesium and postoperative AKI. Supplemental Tables 1–3 show that significant associations between high early postoperative serum magnesium level and AKI remained in the three sensitivity analyses. An early increase in serum magnesium after cardiac surgery from preoperative baseline levels (per 1 mg/dL increase in ΔMg) was independently associated with a higher risk of postoperative AKI (adjusted OR, 1.18; 95% CI, 1.05–1.33; *p* = .006; Supplemental Table 4). In categorical analysis, individuals in the top serum magnesium quartile (Q4) had a 47% higher risk of any-stage AKI compared with those in the lowest quartile (Q1) (adjusted OR, 1.47; 95% CI, 1.16–1.86; *p* for trend <.001; Supplemental Table 4). However, there were no significant associations between preoperative serum magnesium (per 1 mg/dL increase) and postoperative AKI (adjusted OR, 1.10; 95% CI, 0.95–1.86; *p* = .223; Supplemental Table 5). Also, there was no trend toward higher risk of AKI for increasing quartiles of preoperative serum magnesium (*p* for trend=.058; Supplemental Table 5). Of note, the last two sensitivity analyses only included 2505 cardiac surgery patients as preoperative serum magnesium values were not available in the excluded patients from the main cohort.

In addition, the potential effect of use of loop diuretics was tested. Preoperative loop diuretics were given in 2.3% of the study cohort while loop diuretics were used in 4.0% of patients after surgery but before the first postoperative serum magnesium measurement. The association between serum magnesium and AKI remained statistically significant after accounting for the use of loop diuretics as covariates in the multivariable logistic regression model (Supplemental Table 6). Another *post hoc* analysis excluding patients with no available preoperative serum creatinine measurements confirmed the findings from the main analysis (Supplemental Table 7). Lastly, early postoperative serum potassium was not a significant predictor of AKI (adjusted OR, 1.03 per 1 mmol/L increase; 95% CI, 0.90–1.17; *p*<.692 when serum magnesium considered as a continuous variable; and adjusted OR, 1.03 per 1 mmol/L increase; 95% CI, 0.91–1.17; *p*<.641 when serum magnesium considered as a categorical variable) and the results were consistent with the main analysis with early postoperative serum potassium as an additional potential confounding variable (Supplemental Table 8).

### Subgroup and exploratory analyses

In subgroup analyses, the association of early postoperative serum magnesium with postoperative AKI was more pronounced in female patients, those with hypertension, and those receiving magnesium supplementation (Supplemental Table 9). There was no significant effect modification of the associations between serum magnesium and postoperative AKI by patient age, ethnicity, admission type, surgery type, baseline eGFR, and comorbidities including CHF, diabetes, and CKD.

We further explored the association between early postoperative magnesium supplementation and any-stage AKI. When serum magnesium levels were stratified into four grouped by quartiles, magnesium supplementation initiated within 24 h after surgery was associated with a 28% lower risk of AKI in patients in Q1 (adjusted OR, 0.72; 95% CI, 0.56–0.92; *p* = .010) and a 53% higher risk of AKI in those in Q2 (adjusted OR, 1.53; 95% CI, 1.12–2.09; *p* = .008; Supplemental Figure 2). There were no significant associations between magnesium supplementation and postoperative AKI in subjects in Q3 and Q4.

## Discussion

In this multicenter retrospective cohort study of 6124 adult patients undergoing cardiac surgery, higher early postoperative serum magnesium levels were associated with a higher risk of postoperative AKI in a multivariable logistic regression analysis. Also, patients with higher serum magnesium had a significantly increased odds of new-onset postoperative RRT. We did not observe an association between early postoperative serum magnesium and in-hospital mortality. Interestingly, the effect of higher serum magnesium on postoperative AKI was more pronounced in females, those with hypertension, and those given early postoperative magnesium supplementation.

In our cohort, the incidence of cardiac surgery-associated AKI was 57.1%, which was relatively high. This could be the result of using both serum creatinine and urine output criteria for the diagnosis of postoperative AKI in our study. In the sensitivity analysis with AKI diagnosed only by the serum creatinine criteria, the incidence of AKI in our cohort fell significantly to 30.3%, similar to the rate reported in the literature [3].

Our findings that higher serum magnesium was associated with significantly higher risks of AKI as well as new-onset RRT were consistent with previous studies of different patient populations. In a single-center retrospective cohort study of 9241 hospitalized adult patients, hypermagnesemia (serum magnesium >2.3 mg/dL) at hospital admission was associated with increased odds of in-hospital AKI (adjusted OR, 1.42; 95% CI, 1.11–1.81) compared with those with normal serum magnesium levels (1.9–2.1 mg/dL) [20]. Another cohort study retrospectively investigated 1685 patients with COVID-19 and found patients who had hypermagnesemia, defined as mean serum magnesium level greater than 2.4 mg/dL during the entire hospital stay, had higher incidences of both AKI (65% vs. 50%; *p* = .001) and RRT (18% vs. 5%; *p* = .001) compared to those without hypermagnesemia [21]. Moreover, a recent retrospective study of 3669 critically ill children found that hypermagnesemia at ICU admission was independently associated with increased odds of AKI (adjusted OR, 1.52; 95% CI, 1.27–1.82) [22].

Prior studies have indicated that hypermagnesemia could be caused by the disruption of magnesium excretion in the kidney after AKI [20,22–25]. In a sensitivity analysis of our primary outcome, we showed that an increase of serum magnesium from preoperative baseline levels was also independently associated with a higher risk of postoperative AKI. It was unclear whether this increase of serum magnesium in the perioperative period was the result of the development of AKI or excessive magnesium supplementation [26]. Of note, a subgroup analysis of the interaction between magnesium supplementation and serum magnesium in our study showed that patients receiving early postoperative magnesium supplementation had an increased risk of AKI than those without. We also reported that magnesium supplementation was associated with higher risk of AKI in patients with relatively normal early postoperative serum magnesium levels (2.0–2.2 mg/dL) (adjusted OR, 1.53; 95% CI, 1.12–2.09). Although the association between serum magnesium and AKI cannot be assumed to be causal due to the observational nature of our study, early high postoperative serum magnesium level could be an early warning sign of the development of AKI. As renal injury occurs early after surgery, serum magnesium level might start to rise before its diagnosis by either serum creatinine or urine output criteria due to decreased urinary excretion of magnesium. As previous studies suggested that female patients were more likely to develop hypomagnesemia [27], our finding that the association of higher early postoperative serum magnesium with postoperative AKI was more pronounced in female patients could be the results that bigger increase of serum magnesium level in females compared to males increased the risk of having AKI. In subgroup analysis, we also found that the association of higher early postoperative serum magnesium with postoperative AKI was more pronounced in patients with hypertension. This could be the result that hypertensive patients with higher postoperative serum magnesium levels had higher risk of renal hypoperfusion as higher serum magnesium was reported to be associated with lower blood pressure [14].

The association between hypomagnesemia and renal injury after cardiac surgery has been explored previous studies. A retrospective cohort study of 539 cardiac surgery patients found that lower preoperative serum magnesium was associated with increased odds for developing postoperative AKI (adjusted OR, 1.56; 95% CI, 1.18–2.07, per 0.1 mg/dL increase). Additionally, in the categorical analysis, preoperative serum magnesium less than 1.61 mg/dL was independently associated with a threefold higher risk of postoperative AKI [15]. In another similar study of 9766 patients undergoing cardiac surgery, preoperative ionized magnesium in the lowest quartile (Q1) was associated with 53% higher risk of AKI compared with the top quartile (Q4) [28]. This study also showed that preoperative hypomagnesemia (serum ionized magnesium level <1.09 mg/dL) was independently associated with AKI (adjusted OR, 1.39; 95% CI, 1.10–1.77) and RRT (adjusted OR, 1.67; 95% CI, 1.02–2.72). In the primary and sensitivity analyses of our study, however, neither preoperative nor early postoperative low serum magnesium was associated with postoperative AKI. Of note, we did show a protective effect of magnesium supplementation against AKI (adjusted OR, 0.72; 95% CI, 0.56–0.92) in patients with low early postoperative serum magnesium level (<2.0 mg/dL) but not in others with higher values. These mixed results warrant further investigation regarding the effects of low serum magnesium and magnesium supplementation on cardiac surgery-associated AKI.

Although our results stem from a multicenter large cohort of cardiac surgery patients with ethnical and racial diversity, several limitations should be acknowledged. First, the effects of intraoperative serum magnesium, intraoperative magnesium supplementation, use of cardiopulmonary bypass on postoperative AKI could not be assessed as these data were not available in the eICU database. Second, our results might suffer from selection bias as only patients with available serum magnesium in the first 24 h after surgery were included in the primary analysis. Third, ionize serum magnesium and total magnesium body stores were not assessed in our study and should be measured in future studies for better understanding of the effect of magnesium imbalance on outcomes [6,29].

## Conclusions

In conclusion, we demonstrate a higher early postoperative serum magnesium level was independently associated with cardiac surgery-associated AKI as well as new-onset postoperative RRT. Our findings should be confirmed in interventional trials to further determine the effect of magnesium imbalance on postoperative AKI.

## Supplementary Material

Supplemental Material

## Data Availability

All data generated or analyzed during this study are included in this article and its supplementary material files. Further enquiries can be directed to the corresponding author.
