# Supplementary material for: Association of early postoperative serum magnesium with acute kidney injury after cardiac surgery
Source: Ren Fail. 2023 Feb 2;45(1):2170244. doi: 10.1080/0886022X.2023.2170244 (PMC9897740; doi:10.1080/0886022X.2023.2170244)
Supplement: Supplemental Material [file IRNF_A_2170244_SM6691.pdf]

Appendix A. Supplementary data

Supplementary Table 1. Association of early postoperative serum magnesium with acute kidney injury after cardiac surgery assessed by Cox proportional hazard regression.

Supplementary Table 2. Association of early postoperative serum magnesium with cardiac surgery-associated acute kidney injury according to serum creatinine criteria only.

Supplementary Table 3. Association of early postoperative serum magnesium with cardiac surgery-associated acute kidney injury in patients after multiple imputation.

Supplementary Table 4. Association of ΔMg with cardiac surgery-associated acute kidney injury.

Supplementary Table 5. Association of preoperative serum magnesium with cardiac surgery-associated acute kidney injury.

Supplementary Table 6. Association of early postoperative serum magnesium with cardiac surgery-associated acute kidney injury further adjusted for use of loop diuretics.

Supplementary Table 7. Association of early postoperative serum magnesium with cardiac surgery-associated acute kidney injury excluding patients without preoperative serum creatinine.

Supplementary Table 8. Association of early postoperative serum magnesium with cardiac surgery-associated acute kidney injury further adjusted for early postoperative serum potassium.

Supplementary Table 9. Subgroup analyses of the associations between early postoperative serum magnesium levels and acute kidney injury in logistic regression models.

Supplementary Fig 1. Distribution of early postoperative serum magnesium.

Supplementary Fig 2. Multivariable logistic regression model assessing the association of early postoperative magnesium supplementation with AKI. Model adjusted for age, sex, ethnicity, body mass index, admission type, surgery type, comorbidities, baseline hemoglobin, baseline serum creatinine, baseline eGFR, SOFA score, and APACHE IV score.

Information Classification: General

Supplementary Table 1. Association of early postoperative serum magnesium with acute kidney injury after cardiac surgery assessed by Cox proportional hazard regression.

Model	HR (95% CI) per 1mg/dL increase in serum magnesium	*P* value	HR (95% CI)				*P* trend
			Q1 (< 2.0 mg/dL)	Q2 (2.0 –< 2.3mg/dL)	Q3 (2.3 – < 2.7 mg/dL)	Q4 (≥ 2.7 mg/dL)	
N events / N participants	3,498 / 6,124	N/A	701 / 1,386	812 / 1,522	978 / 1,675	1,007 / 1,541	N/A
Univariable	1.31 (1.24 to 1.39)	<0.001	Referent	1.09 (0.99 to 1.21)	1.27 (1.16 to 1.40)	1.52 (1.38 to 1.67)	<0.001
Multivariable	1.28 (1.20 to 1.36)	<0.001	Referent	1.07 (0.97 to 1.19)	1.21 (1.09 to 1.34)	1.43 (1.29 to 1.58)	<0.001

Hazard ratios (95% confidence intervals) by Cox proportional hazard regression models were adjusted for age, sex, ethnicity, body mass index, admission type, surgery type, comorbidities, baseline hemoglobin, baseline serum creatinine, baseline eGFR, SOFA score, APACHE IV score, and magnesium supplementation. HR, hazard ratio.

Information Classification: General

Supplementary Table 2. Association of early postoperative serum magnesium with cardiac surgery-associated acute kidney injury according to serum creatinine criteria only.

Model	OR (95% CI) per 1mg/dL increase in serum magnesium	*P* value	OR (95% CI)				*P* trend
			Q1 (< 2.0mg/dL)	Q2 (2.0 – < 2.3 mg/dL)	Q3 (2.3 – < 2.7 mg/dL)	Q4 (≥ 2.7 mg/dL)	
N events / N participants	1,857 / 6,124	N/A	383 / 1,386	412 / 1,522	483 / 1,675	579 / 1,541	N/A
Univariable	1.46 (1.32 to 1.61)	<0.001	Referent	0.97 (0.83 to 1.14)	1.06 (0.91 to 1.24)	1.58 (1.35 to 1.84)	<0.001
Multivariable	1.36 (1.22 to 1.52)	<0.001	Referent	0.92 (0.77 to 1.09)	1.00 (0.84 to 1.19)	1.37 (1.15 to 1.63)	<0.001

Odds ratios (95% confidence intervals) by multivariable logistic regression models were adjusted for age, sex, ethnicity, body mass index, admission type, surgery type, comorbidities, baseline hemoglobin, baseline serum creatinine, baseline eGFR, SOFA score, APACHE IV score, and magnesium supplementation. OR, odds ratio.

Information Classification: General

Supplementary Table 3. Association of early postoperative serum magnesium with cardiac surgery-associated acute kidney injury in patients after multiple imputation.

Model	OR per 1mg/dL increase in serum magnesium	*P* value	OR (95% CI)				*P* trend
			Q1 (< 2.0 mg/dL)	Q2 (2.0 – < 2.3 mg/dL)	Q3 (2.3 – < 2.7 mg/dL)	Q4 (≥ 2.7 mg/dL)	
N events / N participants	3,879 / 6,994	N/A	790 / 1,615	891 / 1,734	1,086 / 1,905	1,112 / 1,740	N/A
Univariable	1.53 (1.39 to 1.67)	<0.001	Referent	1.10 (0.96 to 1.26)	1.38 (1.21 to 1.58)	1.85 (1.61 to 2.12)	<0.001
Multivariable	1.46 (1.32 to 1.61)	<0.001	Referent	1.11 (0.95 to 1.29)	1.30 (1.12 to 1.52)	1.72 (1.47 to 2.02)	<0.001

Odds ratios (95% confidence intervals) by multivariable logistic regression models were adjusted for age, sex, ethnicity, body mass index, admission type, surgery type, comorbidities, baseline hemoglobin, baseline serum creatinine, baseline eGFR, SOFA score, APACHE IV score, and magnesium supplementation. OR, odds ratio.

Information Classification: General

Supplementary Table 4. Association of ΔMg with cardiac surgery-associated acute kidney injury.

Model	OR per 1mg/dL increase in ΔMg	*P* value	OR (95% CI)				*P* trend
			Q1 (< -0.4 mg/dL)	Q2 (-0.4 – < -0.1 mg/dL)	Q3 (-0.1 – < 0.5 mg/dL)	Q4 (≥ 0.5 mg/dL)	
N events / N participants	1,400 / 2,505	N/A	312 / 621	301 / 550	350 / 671	437 / 663	N/A
Univariable	1.33 (1.19 to 1.49)	<0.001	Referent	1.20 (0.95 to 1.51)	1.08 (0.87 to 1.34)	1.92 (1.53 to 2.40)	<0.001
Multivariable	1.18 (1.05 to 1.33)	0.006	Referent	0.99 (0.77 to 1.26)	0.88 (0.70 to 1.11)	1.47 (1.16 to 1.86)	<0.001

Odds ratios (95% confidence intervals) by multivariable logistic regression models were adjusted for age, sex, ethnicity, body mass index, admission type, surgery type, comorbidities, baseline hemoglobin, baseline serum creatinine, baseline eGFR, SOFA score, APACHE IV score, and magnesium supplementation. OR, odds ratio.

Information Classification: General

Supplementary Table 5. Association of preoperative serum magnesium with cardiac surgery-associated acute kidney injury.

Model	OR per 1mg/dL increase in serum magnesium	*P* value	OR (95% CI)				*P* trend
			Q1 (≤ 1.9 mg/dL)	Q2 (2.0 – ≤ 2.1 mg/dL)	Q3 (2.2 – < 2.6 mg/dL)	Q4 (≥ 2.6 mg/dL)	
N events / N participants	1,400 / 2,505	N/A	427 / 730	282 / 526	316 / 583	375 / 666	N/A
Univariable	0.99 (0.87 to 1.14)	0.933	Referent	0.82 (0.65 to 1.03)	0.84 (0.67 to 1.05)	0.91 (0.74 to 1.13)	0.299
Multivariable	1.10 (0.95 to 1.27)	0.223	Referent	0.84 (0.66 to 1.07)	0.83 (0.65 to 1.04)	1.09 (0.87 to 1.37)	0.058

Odds ratios (95% confidence intervals) by multivariable logistic regression models were adjusted for age, sex, ethnicity, body mass index, admission type, surgery type, comorbidities, baseline hemoglobin, baseline serum creatinine, baseline eGFR, SOFA score, APACHE IV score, and magnesium supplementation. OR, odds ratio.

Information Classification: General

Supplementary Table 6. Association of early postoperative serum magnesium with cardiac surgery-associated acute kidney injury further adjusted for use of loop diuretics.

Model	OR per 1mg/dL increase in serum magnesium	*P* value	OR (95% CI)				*P* trend
			Q1 (≤ 1.9 mg/dL)	Q2 (2.0 – ≤ 2.1 mg/dL)	Q3 (2.2 – < 2.6 mg/dL)	Q4 (≥ 2.6 mg/dL)	
N events / N participants	3,498 / 6,124	N/A	701 / 1,386	812 / 1,522	978 / 1,675	1,007 / 1,541	N/A
Univariable	1.52 (1.38–1.68)	<0.001	Referent	1.12(0.97-1.29)	1.37(1.19-1.58)	1.84(1.59-2.14)	<0.001
Multivariable	1.45 (1.32–1.62)	<0.001	Referent	1.11(0.95-1.29)	1.30(1.11-1.51)	1.71(1.45-2.00)	<0.001

Odds ratios (95% confidence intervals) by multivariable logistic regression models were adjusted for age, sex, ethnicity, body mass index, admission type, surgery type, comorbidities, baseline hemoglobin, baseline serum creatinine, baseline eGFR, SOFA score, APACHE IV score, magnesium supplementation, preoperative use of loop diuretics, and postoperative use of loop diuretic. OR, odds ratio.

Information Classification: General

Supplementary Table 7. Association of early postoperative serum magnesium with cardiac surgery-associated acute kidney injury excluding patients without preoperative serum creatinine.

Model	OR per 1mg/dL increase in serum magnesium	*P* value	OR (95% CI)				*P* trend
			Q1 (≤ 1.9 mg/dL)	Q2 (2.0 – ≤ 2.1 mg/dL)	Q3 (2.2 – < 2.6 mg/dL)	Q4 (≥ 2.6 mg/dL)	
N events / N participants	2,103 / 3,762	N/A	448 / 888	512 / 994	577 / 1,013	566 / 867	N/A
Univariable	1.55 (1.36–1.76)	<0.001	Referent	1.04(0.87-1.25)	1.30(1.08-1.56)	1.84(1.52-2.24)	<0.001
Multivariable	1.49 (1.30–1.70)	<0.001	Referent	1.02(0.84-1.24)	1.20(0.99-1.46)	1.71(1.39-2.10)	<0.001

Odds ratios (95% confidence intervals) by multivariable logistic regression models were adjusted for age, sex, ethnicity, body mass index, admission type, surgery type, comorbidities, baseline hemoglobin, baseline serum creatinine, baseline eGFR, SOFA score, APACHE IV score, and magnesium supplementation. OR, odds ratio.

Information Classification: General

Supplementary Table 8. Association of early postoperative serum magnesium with cardiac surgery-associated acute kidney injury further adjusted for early postoperative serum potassium.

Model	OR per 1mg/dL increase in serum magnesium	*P* value	OR (95% CI)				*P* trend
			Q1 (≤ 1.9 mg/dL)	Q2 (2.0 – ≤ 2.1 mg/dL)	Q3 (2.2 – < 2.6 mg/dL)	Q4 (≥ 2.6 mg/dL)	
N events / N participants	3,496 / 6,120	N/A	699 / 1,384	812 / 1,522	978 / 1,673	1,007 / 1,541	N/A
Univariable	1.52 (1.38–1.68)	<0.001	Referent	1.12(0.97-1.29)	1.37(1.19-1.58)	1.84(1.59-2.14)	<0.001
Multivariable	1.46 (1.32–1.63)	<0.001	Referent	1.11(0.95-1.29)	1.31(1.12-1.52)	1.71(1.47-2.02)	<0.001

Odds ratios (95% confidence intervals) by multivariable logistic regression models were adjusted for age, sex, ethnicity, body mass index, admission type, surgery type, comorbidities, baseline hemoglobin, baseline serum creatinine, baseline eGFR, SOFA score, APACHE IV score, magnesium supplementation, and early postoperative serum potassium. OR, odds ratio.

Information Classification: General

Supplementary Table 9. Subgroup analyses of the associations between early postoperative serum magnesium levels and acute kidney injury in logistic regression models.

Variables	OR per 1mg/dL increase in serum magnesium	*P* for interaction	OR (95% CI)				*P* for interaction
			Q1 (< 2.0 mg/dL)	Q2 (2.0 – < 2.3 mg/dL)	Q3 (2.3 – < 2.7 mg/dL)	Q4 (≥ 2.7 mg/dL)	
**Age, years**		0.244					0.208
≥65	1.52 (1.33 to 1.74)		Referent	1.12 (0.93 to 1.36)	1.35 (1.11 to 1.63)	1.94 (1.57 to 2.38)	
<65	1.38 (1.16 to 1.64)		Referent	1.06 (0.82 to 1.37)	1.21 (0.94 to 1.56)	1.44 (1.11 to 1.88)	
**Sex**		0.494					0.057
Male	1.52 (1.33 to 1.74)		Referent	0.97 (0.81 to 1.17)	1.19 (0.99 to 1.44)	1.71 (1.40 to 2.10)	
Female	1.37 (1.15 to 1.63)		Referent	1.41 (1.07 to 1.87)	1.51 (1.15 to 1.99)	1.74 (1.32 to 2.30)	
**Ethnicity**		0.404					0.318
White	1.51 (1.34 to 1.71)		Referent	1.06 (0.90 to 1.26)	1.25 (1.06 to 1.47)	1.71 (1.44 to 2.04)	
Other	1.22 (0.96 to 1.54)		Referent	1.27 (0.84 to 1.90)	1.60 (1.06 to 2.40)	1.65 (1.09 to 2.50)	
**Admission type**		0.246					0.333
Non-elective	1.19 (0.76 to 1.84)		Referent	0.74 (0.39 to 1.43)	1.34 (0.68 to 2.64)	1.24 (0.61 to 2.52)	
Elective	1.49 (1.33 to 1.66)		Referent	1.13 (0.96 to 1.32)	1.30 (1.11 to 1.52)	1.76 (1.49 to 2.08)	
**Surgery type**		0.720					0.898
CABG only	1.44 (1.25 to 1.66)		Referent	1.06 (0.86 to 1.30)	1.24 (1.01 to 1.51)	1.69 (1.36 to 2.10)	
Valve only	1.44 (1.20 to 1.73)		Referent	1.07 (0.83 to 1.40)	1.34 (1.02 to 1.76)	1.74 (1.32 to 2.30)	
Combined	1.64 (1.15 to 2.33)		Referent	1.18 (0.67 to 2.06)	1.34 (0.76 to 2.36)	1.71 (0.97 to 3.02)	
**CHF**		0.551					0.441
Yes	1.69 (1.29 to 2.20)		Referent	0.98 (0.69 to 1.41)	1.09 (0.74 to 1.60)	1.95 (1.31 to 2.90)	
No	1.44 (1.29 to 1.62)		Referent	1.11 (0.94 to 1.32)	1.32 (1.12 to 1.57)	1.71 (1.43 to 2.04)	
**Hypertension**		0.468					0.090
Yes	1.55 (1.36 to 1.78)		Referent	1.21 (1.00 to 1.46)	1.35 (1.12 to 1.63)	2.05 (1.67 to 2.51)	
No	1.34 (1.13 to 1.59)		Referent	0.90 (0.68 to 1.17)	1.16 (0.89 to 1.51)	1.28 (0.98 to 1.68)	
**Diabetes**		0.749					0.588
Yes	1.57 (1.29 to 1.91)		Referent	1.21 (1.00 to 1.46)	1.35 (1.12 to 1.63)	2.05 (1.67 to 2.51)	
No	1.34 (1.13 to 1.59)		Referent	1.11 (0.92 to 1.34)	1.25 (1.04 to 1.52)	1.76 (1.44 to 2.14)	
**Chronic kidney disease**		0.316					0.416
Yes	1.30 (0.87 to 1.95)		Referent	1.02 (0.61 to 1.71)	1.48 (0.82 to 2.67)	1.42 (0.78 to 2.57)	
No	1.48 (1.33 to 1.66)		Referent	1.09 (0.92 to 1.28)	1.27 (1.08 to 1.49)	1.74 (1.47 to 2.06)	
**Baseline eGFR**		0.119					0.406
<60	1.33 (1.02 to 1.75)		Referent	1.18 (0.77 to 1.81)	1.60 (1.02 to 2.50)	1.72 (1.11 to 2.67)	
≥60	1.49 (1.32 to 1.67)		Referent	1.07 (0.91 to 1.26)	1.24 (1.05 to 1.47)	1.71 (1.44 to 2.04)	
**Magnesium supplement**		0.025					0.002
Yes	2.10 (1.39 to 3.19)		Referent	1.08 (0.83 to 1.41)	1.43 (1.10 to 1.86)	1.64 (1.24 to 2.18)	
No	1.43 (1.26 to 1.62)		Referent	0.97 (0.82 to 1.15)	1.23 (1.04 to 1.46)	1.59 (1.34 to 1.88)	

Odds ratios (95% confidence intervals) by multivariable logistic regression models were adjusted for age, sex, ethnicity, body mass index, admission type, surgery type, comorbidities, baseline hemoglobin, baseline serum creatinine, baseline eGFR, SOFA score, APACHE IV score, and magnesium supplementation. CHF, congestive heart failure; OR, odds ratio.

Information Classification: General

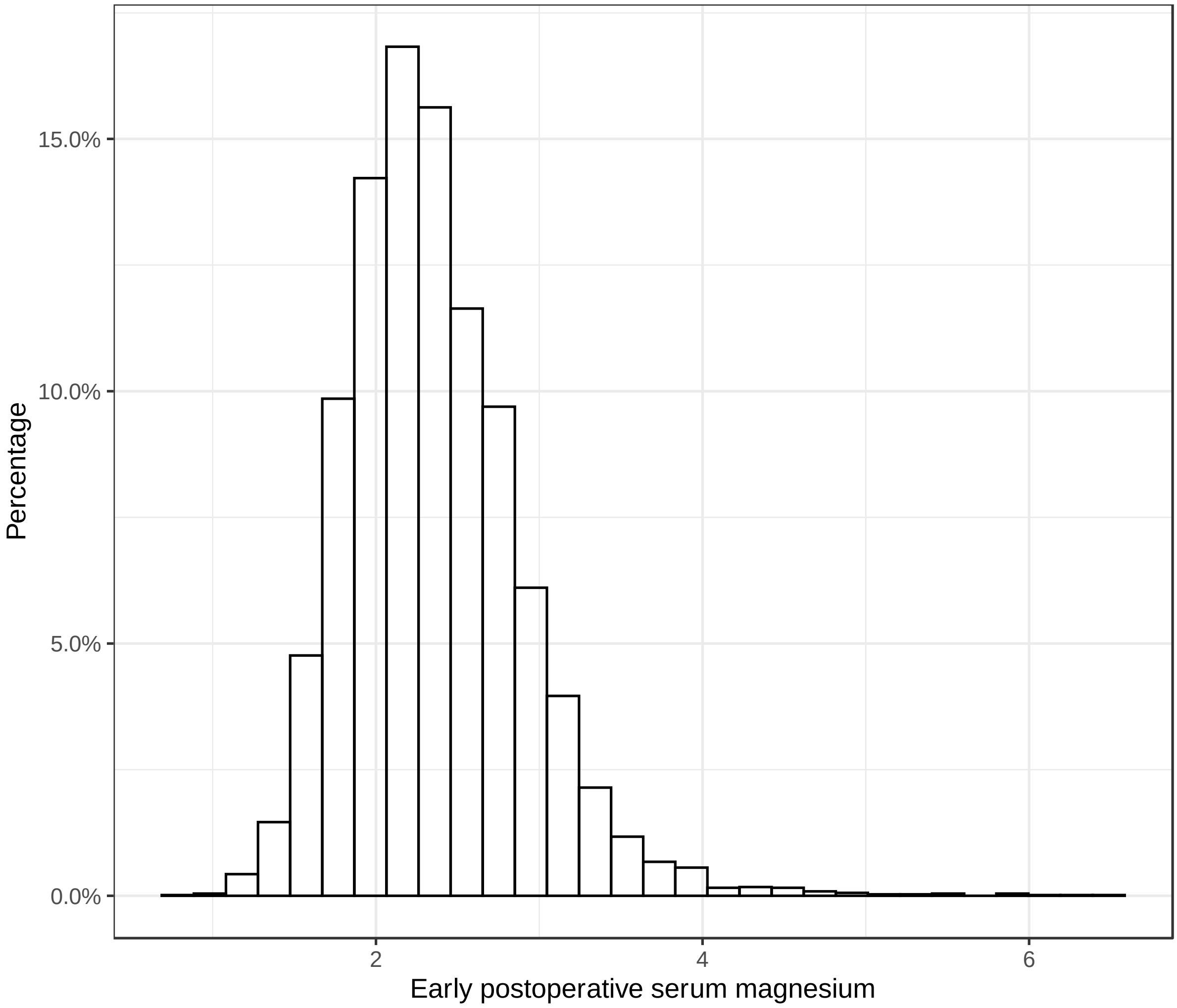

Supplementary Fig. 1. Distribution of early postoperative serum magnesium.

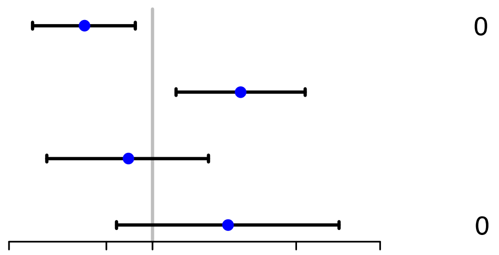

Supplementary Fig. 2. Multivariable logistic regression model assessing the association of early postoperative magnesium supplementation with AKI.
